# Inhibiting CXCR6 promotes senescence of activated hepatic stellate cells with limited proinflammatory SASP to attenuate hepatic fibrosis

**DOI:** 10.1515/biol-2025-1151

**Published:** 2025-08-08

**Authors:** Liqin Sheng, Yiming Wu, Fei Shen, Chenzhou Xu

**Affiliations:** Department of Infectious Diseases, Haiyan People’s Hospital, Jiaxing, Zhejiang, China; Department of Gastroenterology, The First Hospital of Jiaxing, Affiliated Hospital of Jiaxing University, Jiaxing, Zhejiang, China; Department of Gastroenterology, The First Hospital of Jiaxing, Affiliated Hospital of Jiaxing University, 1882 Zhonghuan South Road, Nanhu District, Jiaxing, Zhejiang, China

**Keywords:** hepatic stellate cells, senescence, SASP, CXCR6, hepatic fibrosis

## Abstract

This study investigates the previously unexplored role of CXC chemokine receptor 6 (CXCR6) in hepatic fibrosis, where excessive extracellular matrix deposition by activated hepatic stellate cells (aHSCs) drives disease progression. Through analysis of gene expression omnibus datasets and human fibrotic liver samples, we identified significant CXCR6 upregulation, subsequently validated in murine fibrosis models. Using quantitative real-time polymerase chain reaction, western blotting, and immunohistochemistry, we demonstrated that CXCR6 silencing *in vitro* promoted aHSC senescence – as confirmed by senescence-associated β-galactosidase staining and Cell Counting Kit-8 assays – while simultaneously restricting the pro-inflammatory senescence-associated secretory phenotype (SASP). Mechanistically, the enzyme-linked immunosorbent assay revealed this process involves modulation of the interleukin-1 alpha/nuclear factor-kappa beta feedback loop. Our findings position CXCR6 inhibition as a promising therapeutic strategy that uniquely targets both fibrogenesis (through hepatic stellate cell senescence induction) and inflammation (via SASP regulation) in hepatic fibrosis.

## 1 Introduction

Hepatic fibrosis, a pathological response to chronic liver injury characterized by excessive deposition of the extracellular matrix (ECM), disrupts the normal liver architecture and serves as a precursor to cirrhosis [1]. This condition results from prolonged liver damage induced by factors such as hepatitis virus infection, alcohol abuse, or nonalcoholic steatohepatitis [2]. Hepatic cirrhosis, a consequence of advanced hepatic fibrosis, is the 11th leading cause of death worldwide, underscoring the urgent need for effective therapies to alleviate the health burden associated with cirrhosis. The overproduction of ECM in hepatic fibrosis is primarily driven by fibrogenic myofibroblasts [3,4]. Hepatic stellate cells (HSCs), the main progenitors of these myofibroblasts, play a pivotal role in the fibrogenic process. Under normal physiological conditions, HSCs exist in a quiescent state and store lipid droplets rich in vitamin A. When exposed to profibrotic stimuli, quiescent HSCs become activated and transdifferentiate into myofibroblast-like cells, termed activated HSCs (aHSCs). These aHSCs acquire various properties, including enhanced ECM synthesis, increased proliferation, and activation of inflammatory signaling pathways [5]. Furthermore, inhibiting aHSCs through phenotypic reversion, induction of apoptosis, or inhibition of glycolytic and contractile functions has been demonstrated to mitigate hepatic fibrosis [6,7,8,9]. Recent studies have also emphasized HSC senescence as a promising therapeutic target for hepatic fibrosis [10].

Cellular senescence is a physiological process in which proliferating cells enter a permanent state of cell cycle arrest, preventing re-entry into the cell cycle [11]. This process is characterized by a reduction in genes involved in proliferation and ECM synthesis, alongside an increase in inflammatory cytokine production and other mediators that influence the microenvironment and immune responses [12]. In aHSCs, senescence leads to decreased ECM synthesis, increased ECM-degrading enzyme activity, and enhanced immune surveillance [13]. Consequently, inducing senescence in aHSCs may serve as a potential therapeutic strategy for hepatic fibrosis. Moreover, cellular senescence is frequently associated with a senescence-associated secretory phenotype (SASP), which involves the secretion of chemokines, growth factors, and other proteins [14]. However, the precise molecular mechanisms regulating SASP in hepatic fibrosis remain poorly understood. Identifying and targeting key regulators of SASP may provide valuable insights into therapeutic strategies that exploit the beneficial effects of senescence in treating hepatic fibrosis.

CXC chemokine receptor 6 (CXCR6), a G-protein-coupled receptor with seven transmembrane domains, belongs to the CXC chemokine receptor family [15]. CXCL16, the sole ligand eliciting cytokine secretion via the nuclear factor-κB (NF-κB) and extracellular signal-regulated kinase/mitogen-activated protein kinase pathways thereby influences the immune microenvironment [16,17,18]. In hepatocellular carcinoma, CXCR6 plays a crucial role in regulating hepatocyte senescence through immune surveillance, ultimately suppressing hepatocarcinogenesis [19]. Similarly, HSC activation, along with the associated alterations in inflammatory signaling and the immune microenvironment, is closely associated with CXCR6 activation [20]. Consequently, CXCR6 may emerge as a promising therapeutic target for modulating HSC behavior.

In this study, CXCR6 was found to be upregulated in hepatic fibrosis. Inhibition of CXCR6 promoted cellular senescence in aHSCs, unveiling a novel mechanism underlying the anti-fibrotic effects of CXCR6 modulation.

## 2 Materials and methods

### 2.1 Patient specimens

Paraffin-embedded liver sections from human tissues were obtained from the Affiliated Hospital of Jiaxing University between 2020 and 2023. The fibrotic liver specimens used in our study were indeed obtained from patients with confirmed hepatic fibrosis, while the normal liver tissues were collected from the non-tumor regions of hepatic hemangioma patients. These control tissues showed no signs of fibrosis or other liver injuries.


**Informed consent:** Informed consent was obtained from all individuals included in this study.
**Ethical approval:** The research related to human use complied with all the relevant national regulations, institutional policies, and in accordance with the tenets of the Helsinki Declaration, and was approved by the Ethics Committee of The First Hospital of Jiaxing (Jiaxing, China) (study/trial no.: 2013-017).

### 2.2 Public data collection

Datasets GSE171294, GSE84044, GSE14323, GSE25097, and GSE6764 were retrieved from the gene expression omnibus (http://www.ncbi.nlm.nih.gov/) and used to analyze the differences in CXCR6 expression between normal controls and patients with liver fibrosis.

### 2.3 Animal model and experimental protocols

Twenty male ICR mice (18–20 g; 6–8 weeks old) were purchased from the Animal Center of Jiaxing University and housed in a pathogen-free environment.

Hepatic fibrosis was induced using two methods: common bile duct ligation (BDL) and intraperitoneal injections of carbon tetrachloride (CCl_4_). For BDL, mice were anesthetized with a 40 mg/kg intraperitoneal injection of 3% sodium pentobarbital under sterile conditions. A midline abdominal incision was made, and the common bile duct was exposed near the duodenum’s junction. Two ligatures were placed below the bile duct, and a midsection cut was made. The control group underwent identical procedures without ligation. Hepatic fibrosis was induced for 14 days post-surgery, followed by intraperitoneal injections of 0.6 mL/kg body weight CCl_4_ twice a week for 40 days. Liver tissues were harvested at specified time points for further analysis.


**Ethical approval:** The research related to animal use complied with all the relevant national regulations and institutional policies for the care and use of animals (JUMC2021-027).

### 2.4 Cell culture

The human HSC line LX-2 (Cat. BNCC337957) was obtained from Bena Culture Collection (Beijing, China). Cells were cultured in Dulbecco’s modified Eagle’s medium (DMEM) supplemented with 10% fetal bovine serum and 1% penicillin–streptomycin antibiotic and maintained in an incubator at 37°C with 5% CO_2_. For HSC activation, transforming growth factor beta1 (TGF-β1) was added to the LX-2 cells at a concentration of 10 ng/mL for 24 h.

### 2.5 Reagents and antibodies

Etoposide (HY-13629) was sourced from MedChemExpress (Shanghai, China) and used at a concentration of 5 mmol/L *in vitro*. Pyrrolidine dithiocarbamate (PDTC) (HY-18738), also from MedChemExpress, was used at 100 mmol/L. Recombinant human interleukin-1 alpha (rIL-1α) (PHC0011) from Thermo Fisher Scientific (Shanghai, China) was applied at 20 ng/mL *in vitro*. Antibodies against CXCR6 (ab8023), alpha-1 subunit of type I collagen (COL1A1) (ab34710), tumor protein 53 (p53) (ab26), p21 (ab109520), and glyceraldehyde 3-phosphate dehydrogenase (GAPDH) (ab8245) were purchased from Abcam (Shanghai, China). The LaminB1 antibody (12987-1-AP) was obtained from Proteintech (Wuhan, China), while the α-smooth muscle actin (α-SMA) antibody (mAb #19245) was from Cell Signaling Technology (Shanghai, China). Secondary antibodies, including Goat anti-rabbit IgG (H + L), horseradish peroxidase (HRP)-linked (Cat. #7074) and Goat anti-mouse IgG (H + L), HRP-linked (Cat. #7076), were from Cell Signaling Technology (Shanghai, China).

### 2.6 Quantitative real-time polymerase chain reaction (qPCR)

Total RNA from liver tissues and cells was extracted using TRIzol reagent (Thermo Fisher Scientific, Shanghai, China), followed by cDNA synthesis with a first-strand cDNA synthesis kit (Vazyme, Nanjing, China). qPCR was performed using SYBR-Green fluorescence-based assays for signal detection, with GAPDH serving as the internal reference for data normalization. Primer sequences are provided in Table 1.

**Table 1:** Sequence of primers for qPCR

Species	Name	Forward primer (5′–3′)	Reverse primer (5′–3′)
Mouse	*Timp-1*	GCAACTCGGACCTGGTCATAA	CGGCCCGTGATGAGAAACT
Mouse	*Col1a1*	TTCTCCTGGCAAAGACGGAC	CCATCGGTCATGCTCTCTCC
Mouse	*Tgfb*	CTCCCGTGGCTTCTAGTGC	GCCTTAGTTTGGACAGGATCTG
Mouse	*Cxcr6*	GAGTCAGCTCTGTACGATGGG	TCCTTGAACTTTAGGAAGCGTTT
Mouse	*Gapdh*	GGAGAGTGTTTCCTCGTCCC	ACTGTGCCGTTGAATTTGCC
Human	*CXCR6*	GACTATGGGTTCAGCAGTTTCA	GGCTCTGCAACTTATGGTAGAAG
Human	*ACTA2*	AAAAGACAGCTACGTGGGTGA	GCCATGTTCTATCGGGTACTTC
Human	*IL1A*	TGGTAGTAGCAACCAACGGGA	ACTTTGATTGAGGGCGTCATTC
Human	*IL1B*	ATGATGGCTTATTACAGTGGCAA	GTCGGAGATTCGTAGCTGGA
Human	*IL6*	ACTCACCTCTTCAGAACGAATTG	CCATCTTTGGAAGGTTCAGGTTG
Human	*IL8*	AGGACAACAGAGAGGTGTGC	CAGCGGTGCATCAGAATTGAG
Human	*GAPDH*	CTGGGCTACACTGAGCACC	AAGTGGTCGTTGAGGGCAATG

### 2.7 Immunohistochemistry (IHC)

Liver sections were fixed in 4% paraformaldehyde and subsequently washed three times with phosphate buffered saline (PBS). After a 5 min incubation in citrate buffer at 108°C, the slides were exposed to 0.3% hydrogen peroxide. Following that, the sections were incubated with 10% goat serum for 30 min, and the CXCR6 antibody was applied for overnight incubation at 4°C. A secondary antibody was then used for detection.

### 2.8 Histological hematoxylin–eosin (H&E), sirius red, and Masson staining

Liver sections underwent a series of graded alcohol dehydrations, followed by paraffin embedding and sectioning at 5 μm. The sections were stained with Masson’s trichrome, sirius red, and H&E for histological analysis. Ten random fields per slide were captured for Masson and sirius red staining, and the percentage of positively stained areas was quantified using ImageJ software.

### 2.9 Western blotting (WB)

Liver tissues or cells were lysed with RIPA buffer, and protein samples were separated by polyacrylamide gel electrophoresis before being transferred onto polyvinylidene fluoride membranes. After blocking, the membranes were probed with specific antibodies, and protein bands were visualized using the ChemiDoc XRS+ system.

### 2.10 Cell transfection

CXCR6 siRNA (AM16708) was obtained from Thermo Fisher Scientific (Shanghai, China). Transfection was performed using Lipofectamine RNAiMAX Transfection Reagent (Cat. 13778100, Thermo Fisher Scientific) following the manufacturer’s instructions.

### 2.11 Immunohistofluorescence

LX-2 cells were fixed with 4% paraformaldehyde following three washes with PBS. After fixation, the cells were treated with 10% NGS for 1 h and then exposed to 0.5% Triton X-100/PBS for 10 min. The cells were incubated overnight at 4°C with an anti-α-SMA antibody. Subsequently, the cells were incubated with goat anti-rabbit IgG (Alexa Fluor 647-labeled, Cat. 4414S, CST) for 1 h in darkness. The nuclei were stained with 4′,6-diamidino-2-phenylindole (DAPI) solution, and images were captured using fluorescence microscopy.

### 2.12 Colony formation assay

Activated LX-2 cells, treated with or without CXCR6 siRNA, were seeded into a 6-well plate at 500 cells per well. The medium was replaced every 3 days. Colonies were allowed to grow for 2 weeks, followed by fixation with carbinol and staining with 0.1% crystal violet for 20 min.

### 2.13 Cell Counting Kit-8 (CCK-8) assay

Cell proliferation was assessed using the CCK-8 (Cat. HY-K0301, MedChemExpress, Shanghai, China) assay. LX-2 cells pre-treated with TGF-β were seeded into 96-well plates at a density of 1 × 10^3^ cells per 100 μL. Cells were treated with or without CXCR6 siRNA, and after 12, 24, 48, and 72 h of incubation, the medium was replaced with a mixture of 10 μL CCK-8 solution and 90 μL DMEM per well. After 4 h of incubation, absorbance was measured at 450 nm using a microplate reader.

### 2.14 Senescence-associated β-galactosidase (SA-β-Gal) staining

SA-β-Gal staining was performed using the SA-β-Gal staining kit (Cat. C0602, Beyotime, Shanghai, China). Activated LX-2 cells, treated with or without CXCR6 siRNA, were seeded into 24-well plates and allowed to attach overnight. The cells were fixed for 15 min and washed three times with PBS. A total of 250 μL of β-Gal solution was added to each well, and cells were incubated overnight at 37°C as per the protocol. The cells were fixed with 2% formaldehyde/0.2% glutaraldehyde and incubated with X-Gal solution (1 mg/mL, pH 6.0) at 37°C for 16 h (non-hypoxic conditions). Imaging was performed using a Nikon Eclipse Ti microscope (40×). SA-β-Gal+ cells were defined as those showing intense perinuclear blue staining. Three independent researchers counted ≥500 cells/group across five random fields (blinded to treatment).

### 2.15 Enzyme-linked immunosorbent assay (ELISA)

Intracellular IL-1α secretion was measured using the Human IL-1α ELISA Kit (Cat. PI565, Beyotime, Shanghai, China). ELISA was conducted according to the manufacturer’s instructions.

### 2.16 Statistical analysis

Data are presented as mean ± SEM. Statistical analysis was performed using SPSS 20.0 software, and comparisons between two groups were made using a Student’s *t*-test. A *p*-value of less than 0.05 was considered statistically significant.

## 3 Results

### 3.1 CXCR6 is overexpressed in human hepatic fibrosis

**Figure 1:**
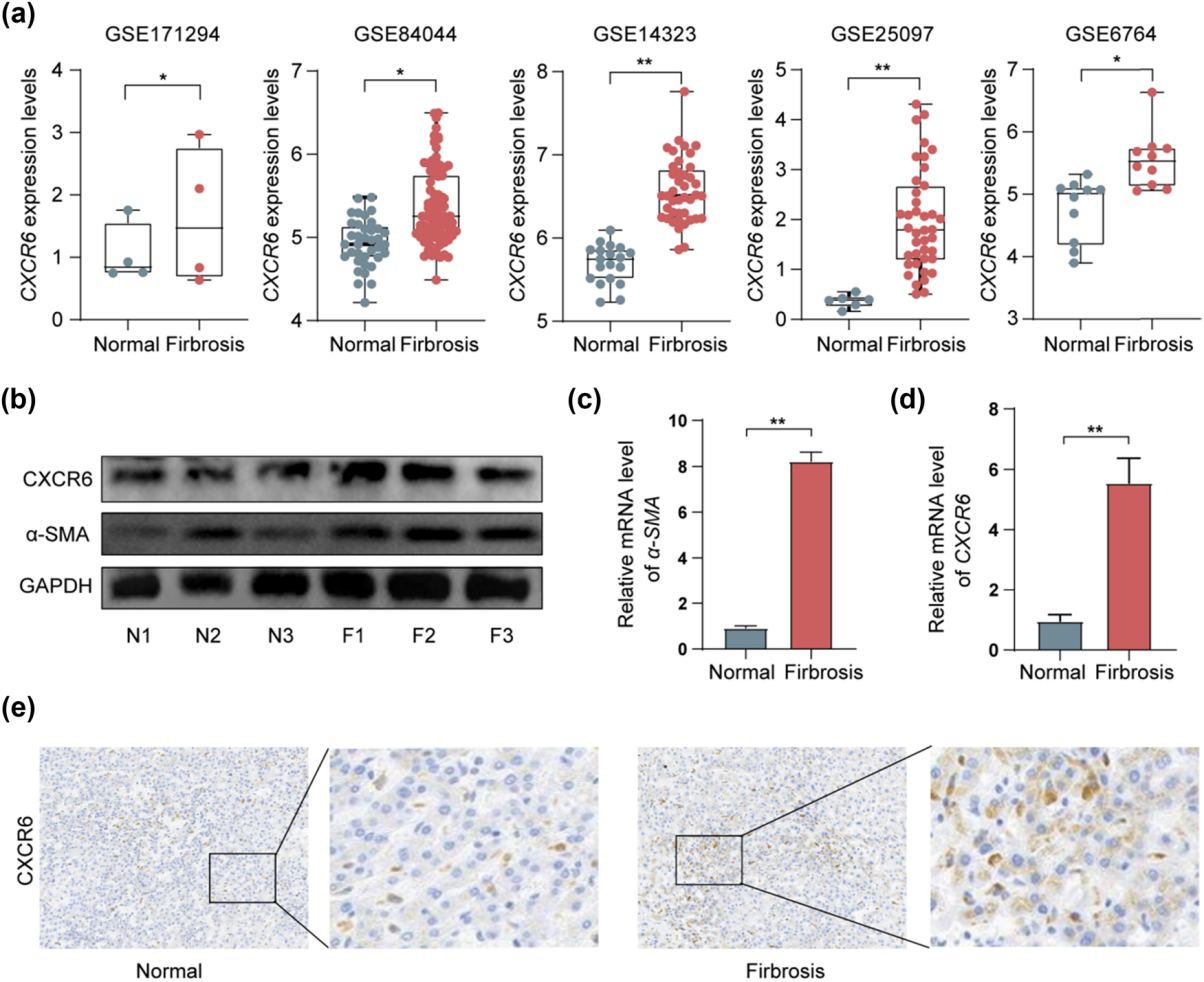
CXCR6 is overexpressed in human hepatic fibrosis. (a) Box plot depicting CXCR6 expression in normal and fibrotic liver tissues across the datasets GSE171294, GSE84044, GSE14323, GSE25097, and GSE6764. (b) WB analysis of CXCR6 and α-SMA proteins in liver fibrosis tissues at different fibrosis stages. (c) and (d) qPCR analysis of *α-SMA* and *CXCR6* mRNA levels in liver fibrosis and normal tissues from human samples. (e) IHC staining showing high CXCR6 expression in liver fibrosis. *p < 0.05, **p < 0.01.

CXCR6 expression was initially analyzed across five publicly available datasets, revealing elevated levels in hepatic fibrosis compared to normal liver tissues (Figure 1a). WB analysis confirmed the enhanced protein expression of both CXCR6 and α-SMA in advanced stages of hepatic fibrosis (Figure 1b). Additionally, qPCR analysis of human liver samples further corroborated the increased mRNA levels of *α-SMA* and *CXCR6* in hepatic fibrosis (Figure 1c and d). IHC analysis also demonstrated a significant upregulation of CXCR6 in fibrotic liver tissues (Figure 1e). These results collectively suggest that CXCR6 is overexpressed in human hepatic fibrosis, highlighting its potential as a therapeutic target.

### 3.2 Enhanced CXCR6 derived from hepatic fibrosis in mouse models

**Figure 2:**
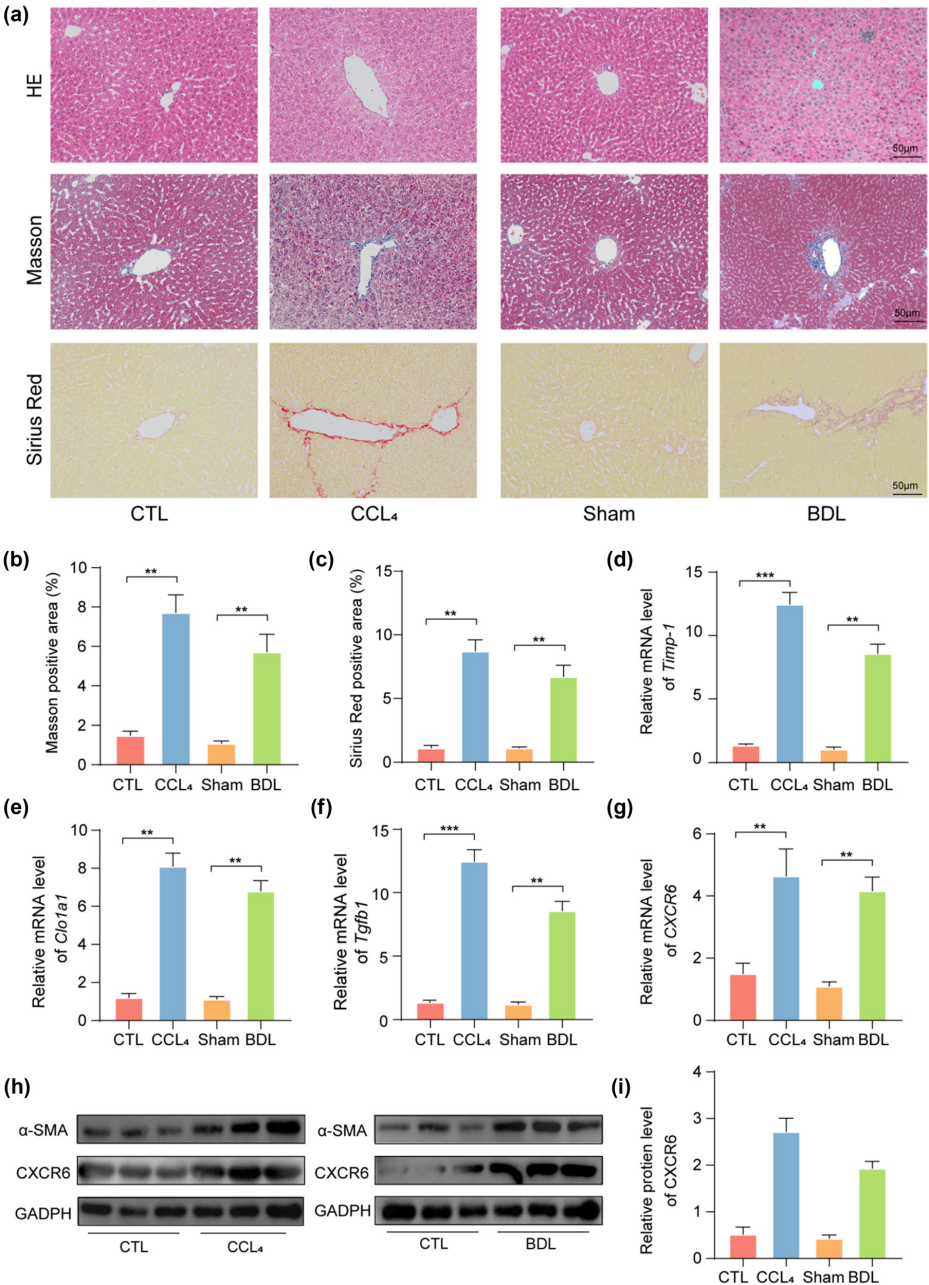
Enhanced CXCR6 expression in hepatic fibrosis mouse models. (a) H&E, Masson, and sirius red staining of liver sections from mice with liver fibrosis. Scale bar: 50 mm; *n* = 6 per group. (b) and (c) Quantification of positive staining for Masson and sirius red (*n* = 6). (d)–(g) mRNA levels (normalized to GAPDH) of *TIMP-1*, *COL1A1*, *TGFB1*, and *CXCR6* in liver fibrosis models (*n* = 6 per group). (h) WB analysis of CXCR6 and α-SMA proteins in liver fibrosis models. (i) Quantification of CXCR6 protein expression in liver fibrosis models. **p < 0.01, ***p < 0.001.

To investigate the role of CXCR6 in hepatic fibrosis, two experimental models were established using intraperitoneal CCl_4_ injections and BDL. As shown in Figure 2a–c, Masson, H&E, and sirius red staining revealed characteristic fibrotic changes in the livers of mice, indicative of ECM accumulation. qPCR analysis revealed an upregulation of tissue inhibitor of metalloproteinase-1 (*TIMP-1*), a known matrix metalloproteinase inhibitor [21], in the liver fibrosis tissues (Figure 2d). Additionally, mRNA levels of fibrosis markers, *COL1A1* and *TGFB1*, were elevated during hepatic fibrosis (Figure 2e and f), alongside an increase in *CXCR6* expression (Figure 2g). WB analysis consistently showed higher protein levels of α-SMA and CXCR6 in the hepatic fibrosis models (Figure 2h and i). These results collectively demonstrate the upregulated expression of CXCR6 in hepatic fibrosis in mouse models.

### 3.3 CXCR6 inhibition promotes cellular senescence of aHSCs

**Figure 3:**
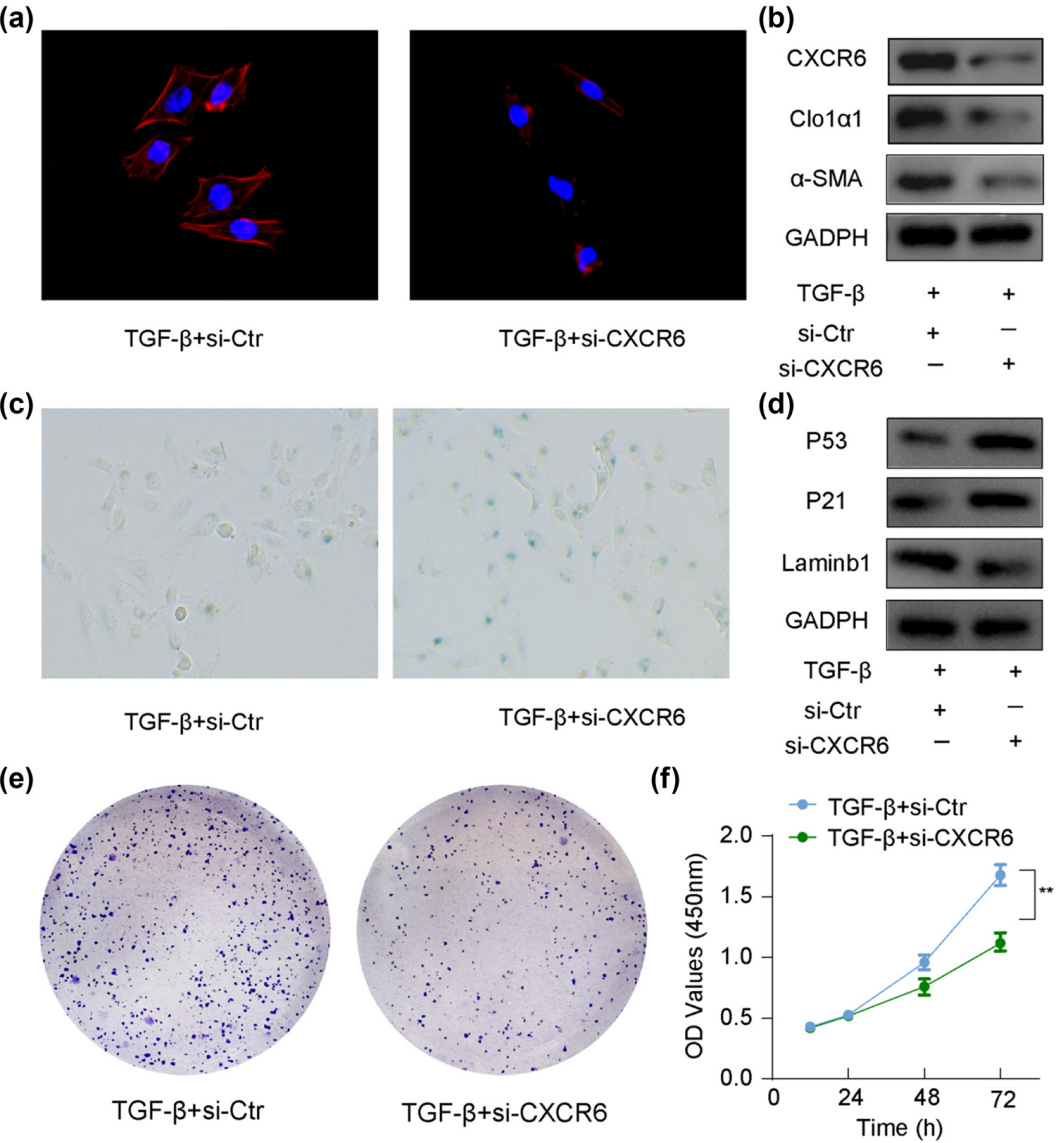
CXCR6 inhibition induces cellular senescence in aHSCs. (a) Representative images of immunofluorescence co-staining for α-SMA (red) and DAPI (blue) in aHSCs treated with or without CXCR6 siRNA. (b) WB analysis showing reduced protein levels of CXCR6, COL1α1, and α-SMA in aHSCs treated with CXCR6 siRNA. (c) SA-β-Gal staining in aHSCs treated with or without CXCR6 siRNA. (d) WB analysis of senescence-related proteins in aHSCs treated with or without CXCR6 siRNA. (e) Colony formation assays in aHSCs treated with or without CXCR6 siRNA. (f) Cell viability assessment using the CCK-8 assay in aHSCs treated with or without CXCR6 siRNA. **p < 0.01.

Cellular senescence results in an irreversible cessation of cell growth, characterized by increased SA-β-gal activity and suppressed cell proliferation [22]. Senescent aHSCs exhibit reduced ECM secretion, and inducing HSC senescence has been proposed as a potential strategy to alleviate hepatic fibrosis [23]. To further investigate the role of CXCR6 in HSC senescence, LX-2 cells were activated with TGF-β1 and subsequently treated with CXCR6 siRNA. Collagen secretion and α-SMA expression are markers of HSC activation [24]. As presented in Figure 3a, CXCR6 knockdown inhibited α-SMA expression, as evidenced by decreased immunofluorescence. In contrast, TGF-β1-treated LX-2 cells exhibited increased protein levels of α-SMA and COL1α1, while CXCR6 inhibition led to downregulation of these markers (Figure 3b, Figure S1b). SA-β-gal staining revealed an elevated number of positive cells in the CXCR6 siRNA-treated group, suggesting that CXCR6 knockdown significantly enhanced SA-β-gal activity (Figure S1c). To explore the mechanisms underlying CXCR6 inhibition-induced HSC senescence, WB analysis was performed to assess the expression of senescent markers p53 and p21. The results demonstrated that CXCR6 knockdown upregulated the expression of p53 and p21, accompanied by a downregulation of lamin-B1 (Figure 3d). Colony formation and CCK-8 assays consistently showed decreased cell proliferation following CXCR6 siRNA treatment (Figure 3e and f, Figure S1a). Together, these results indicate that CXCR6 inhibition promotes senescence in aHSCs.

### 3.4 Inhibition of CXCR6 limits proinflammatory SASP in senescent HSCs

**Figure 4:**
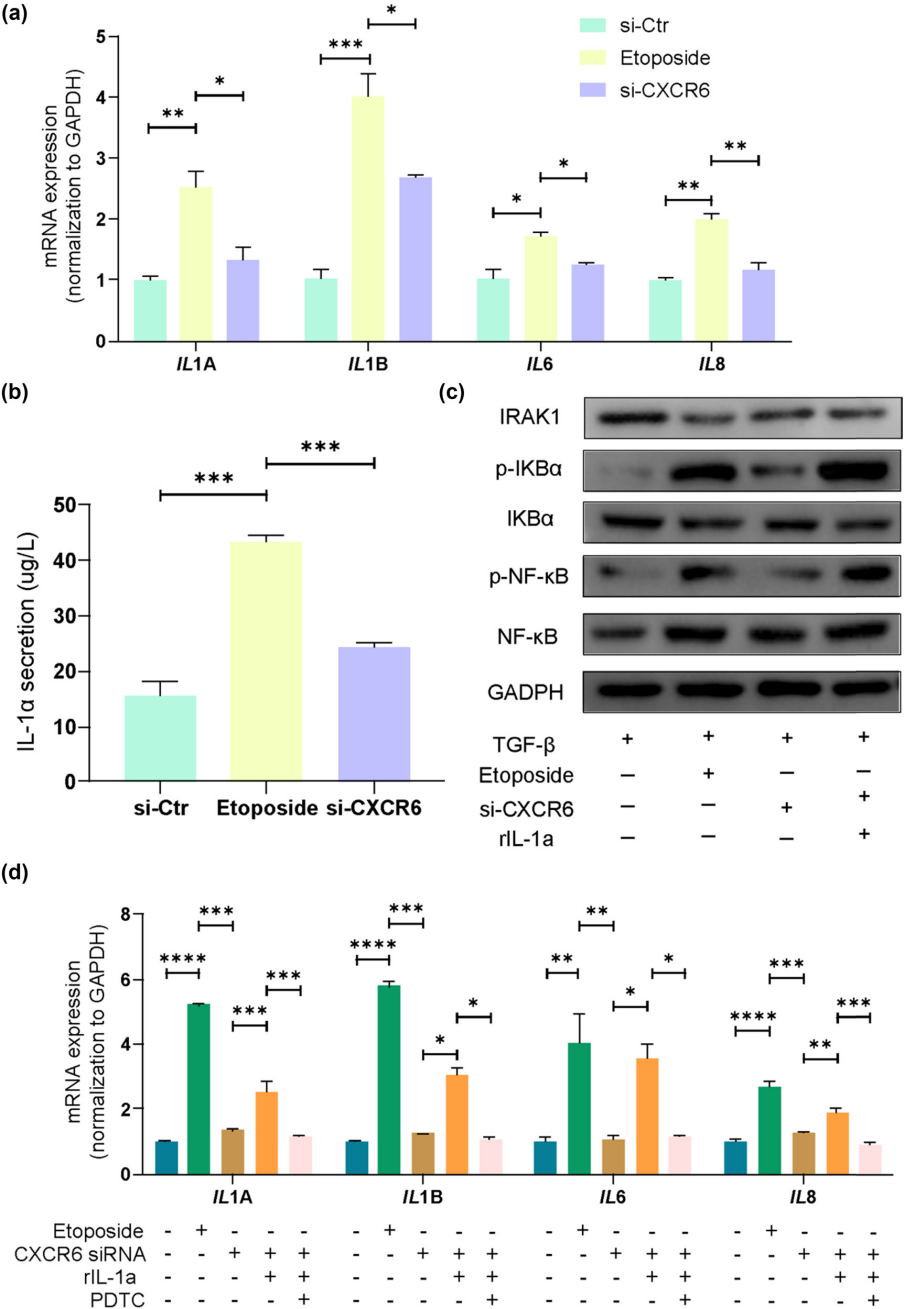
Inhibition of CXCR6 limits proinflammatory SASP in senescent HSCs. (a) qPCR analysis of proinflammatory SASP mRNA levels (IL1A, IL1B, IL6, and IL8) in CXCR6-depleted LX-2 cells (*n* = 3 per group). (b) ELISA of IL-1α secretion (*n* = 3 per group). (c) WB analysis of NF-κB signaling proteins. (d) qPCR analysis of *IL1A*, *IL1B*, *IL6*, and *IL8* in CXCR6-inhibited LX-2 cells treated with rIL-1α and the NF-κB inhibitor PDTC (*n* = 3 per group). *p < 0.05, **p < 0.01, ***p < 0.001, ****p < 0.0001; ns, no significance.

To investigate the relationship between CXCR6 inhibition and the SASP, LX-2 cells were pre-treated with TGF-β1 for activation, followed by exposure to the chemotherapeutic agent etoposide to induce growth arrest and enhance SASP production [25]. PCR analysis revealed that CXCR6-depleted senescent HSCs exhibited a reduction in inflammation-related SASP components, including IL1A, IL1B, IL6, and IL8, compared to the etoposide-treated group (Figure 4a). To explore the mechanisms underlying this effect, IL-1α secretion, a known upstream regulator of SASP and a modulator of IL-6/IL-8 secretion during senescence [26], was measured. ELISA showed that CXCR6 silencing led to a decrease in intracellular IL-1α secretion compared to etoposide treatment (Figure 4b). IL-1α is known to activate the nuclear factor-kappa beta (NF-κB) signaling pathway by binding to its receptor IL1R1, initiating a cascade of signals that culminate in the nuclear translocation of NF-κB [27]. Analysis of IL-1α/NF-κB signaling revealed that CXCR6 inhibition resulted in reduced IL-1α/NF-κB signaling, as evidenced by increased IRAK1 expression and decreased levels of phosphorylated IKBα and phosphorylated NF-κB (Figure 4c). Supplementation with rIL-1α reversed the inhibitory effect of CXCR6 silencing on NF-κB activation, suggesting that CXCR6 depletion hinders the NF-κB signaling pathway by reducing IL-1α levels (Figure 4c). Furthermore, rIL-1α supplementation restored the proinflammatory SASP after CXCR6 siRNA treatment (Figure 4d). Additionally, NF-κB activation is known to upregulate proinflammatory SASP genes such as IL-1α, forming a positive feedback loop involving IL-1α and NF-κB [28]. To validate this, the NF-κB inhibitor PDTC was used in combination with rIL-1α, showing that PDTC attenuated the increase in mRNA levels of IL1A, IL1B, IL6, and IL8 induced by rIL-1α supplementation (Figure 4d). In summary, these results suggest that CXCR6 inhibition limits proinflammatory SASP in senescent HSCs through an IL-1α/NF-κB feedback loop.

## 4 Discussion

Hepatic fibrosis is marked by accumulation of the ECM, which is predominantly synthesized by aHSCs [24]. Targeting senescent aHSCs, in addition to eliminating aHSCs through the inhibition of HSC proliferation and induction of apoptosis, represents a novel therapeutic approach for reversing hepatic fibrosis [29]. The SASP is contingent on the specific senescence trigger and comprises a diverse array of cytokines, growth factors, and proteases secreted by senescent cells [30]. In this study, CXCR6 inhibition promoted senescence in aHSCs, leading to a reduction in the secretion of pro-inflammatory SASP factors. Thus, targeting CXCR6 may offer a potential therapeutic strategy for hepatic fibrosis.

CXCR6, a member of the CXC chemokine receptor family, functions as the receptor for CXCL16 and is involved in cytokine secretion, cell proliferation, and immune regulation [31]. Elevated CXCR6 expression has been observed in various cancer tissues, including prostate cancer [32], pancreatic ductal adenocarcinoma [33], and schwannoma [34], indicating that CXCR6 could be a promising therapeutic target. Our research confirmed that CXCR6 is substantially expressed in patients with advanced hepatic fibrosis, as evidenced by the analysis of public datasets and human fibrotic liver samples. Similar findings were observed in experimental mouse models. Moreover, CXCR6 knockdown alleviated hepatic fibrosis, as indicated by the reduced expression of α-SMA and collagen type I α1 (COL1α1) *in vitro*.

Cellular senescence is characterized by irreversible growth arrest and plays a critical role in various pathological processes, including aging, tumor suppression, and wound healing [35]. Senescent aHSCs exhibit a flattened morphology, along with DNA damage responses such as cell cycle inhibition, high SA-β-gal activity, and SASP production [36]. The mechanisms driving HSC senescence involve various cell stressors, including reactive oxygen species and lysosomal stress [37], as well as the modulation of signaling pathways such as the cyclic GMP-AMP synthase-stimulator of interferon genes (cGAS-STING) pathway and NF-κB signaling. Additionally, metabolic processes associated with deoxycholic acid, produced by gut microbiota, contribute to the progression of HSC senescence [37]. Senescent aHSCs may also express surface ligands that facilitate their clearance by immune cells [38]. In the present study, CXCR6 downregulation induced senescence in aHSCs, as evidenced by increased SA-β-gal activity, decreased α-SMA and COL1α1 expression, and inhibited cell proliferation. Furthermore, studies have demonstrated that p53 overexpression is associated with elevated SA-β-gal activity, increased levels of p21 and p16, and cell cycle arrest [39]. Inhibition of p53 reduces HSC senescence and promotes hepatic fibrosis [40]. Consistent with this, the present study showed that CXCR6 small interfering RNA (siRNA) treatment induced a senescent phenotype in aHSCs, characterized by elevated p53 and p21 expression. Moreover, SASP is a hallmark of cellular senescence. Chronic release of SASP factors can lead to immune cell infiltration, exacerbating local inflammation by inducing paracrine signaling and disrupting tissue homeostasis [41]. Compared to the extensive secretion of SASP factors triggered by etoposide treatment in HSCs, CXCR6 inhibition resulted in reduced pro-inflammatory SASP expression, as evidenced by lower levels of IL-1A, IL-1B, IL-6, and IL-8.

The regulatory mechanisms underlying SASP are complex and involve various cytokines and factors, such as IL-1B, growth-regulated oncogene-α, and neutrophil-activating protein-2, which have been shown to be regulated by the CCAAT/enhancer-binding protein (C/EBP), with C/EBP-β particularly upregulated during oncogene-induced senescence [42]. The SASP can either promote or suppress tumorigenicity depending on the p53 status [43]. A key signaling pathway involved in SASP development is NF-κB, which is primarily activated in response to DNA damage [44,45,46]. Activation of ataxia telangiectasia mutated, a kinase triggered by DNA damage, leads to NF-κB activation through multiple signaling pathways, including p38 mitogen-activated protein kinase (p38MAPK) and retinoic acid-inducible gene I [47]. Additionally, epigenetic changes, such as alterations in sirtuin 6 and high-mobility group box 1 function, can amplify the transcription of inflammation-related genes dependent on NF-κB. Cyclin-dependent kinase inhibitors, such as p14ARF and p16INK4a, act as potent blockers of NF-κB [47]. In the present study, CXCR6-mediated pro-inflammatory SASP via the IL-1α/NF-κB feedback loop.

While aHSCs are unequivocally the primary source of pathological ECM deposition in hepatic fibrosis, the potential contribution of other cell types within the complex hepatic microenvironment, such as liver-derived mesenchymal stem cells (L-MSCs), warrants acknowledgment [48]. L-MSCs exhibit a biphasic role: displaying anti-fibrotic and pro-reparative functions under regenerative conditions and potentially undergoing fibroblastic mesenchymal transition into ECM-producing myofibroblasts under chronic liver injury and sustained profibrotic signaling, notably persistent TGF-β1 stimulation – a key fibrotic driver elevated in our study. Consequently, the profibrotic milieu with high TGF-β1 levels observed here could theoretically facilitate L-MSC pro-fibrotic conversion, positioning them as potential secondary contributors to matrix deposition [49,50]. Nevertheless, aHSCs remain the dominant effector cells responsible for the bulk of pathological scarring. Our findings, demonstrating CXCR6 inhibition effectively targets aHSC senescence and reduces fibrosis, underscore the central importance of modulating aHSC function therapeutically. Further studies are warranted to elucidate the specific contributions and interplay between aHSCs, L-MSCs, and other stromal cells across fibrosis stages and in response to therapies like CXCR6 inhibition.

The strengths of this study are that these *in vivo* data provide strong evidence for the therapeutic potential of CXCR6 inhibition in hepatic fibrosis. Our study has a limitation: the mouse model used in our study may not fully replicate the complex pathophysiology of human hepatic fibrosis. Future research studies would be developed, including the use of more clinically relevant animal models and the exploration of the long-term effects and safety of CXCR6 inhibition.

In conclusion, this study revealed that CXCR6 is upregulated in both human and mouse hepatic fibrosis. Inhibition of CXCR6 promoted HSC senescence and resulted in a reduction of pro-inflammatory SASP through modulation of the IL-1α/NF-κB feedback loop. These findings suggest that CXCR6 inhibition may serve as a potential therapeutic strategy for alleviating hepatic fibrosis.

## Supplementary Material

Supplementary Figure
