# Supplementary material for: Inhibiting CXCR6 promotes senescence of activated hepatic stellate cells with limited proinflammatory SASP to attenuate hepatic fibrosis
Source: Open Life Sci. 2025 Aug 8;20(1):20251151. doi: 10.1515/biol-2025-1151 (PMC12355364; doi:10.1515/biol-2025-1151)
Supplement: Supplementary Figure [file biol-2025-1151-sm.pdf]

Supplementary material

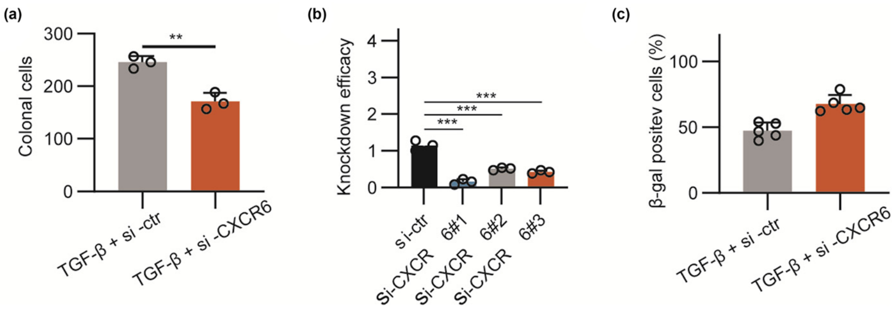

Figure S1: CXCR6 inhibition promotes cellular senescence of aHSCs. (a) Colonal assay for TGF-β+ si-ctr and TGF-β+ si-CXCL6 groups. (b) CXCR6 knockdown efficacy treated withsmall interfering RNA. (c) β-gal assay for TGF-β+ si-ctr and TGF-β+ si-CXCL6 groups.
